# Assessing and Optimizing Large Language Models on Spondyloarthritis Multi-Choice Question Answering: Protocol for Enhancement and Assessment

**DOI:** 10.2196/57001

**Published:** 2024-05-24

**Authors:** Anan Wang, Yunong Wu, Xiaojian Ji, Xiangyang Wang, Jiawen Hu, Fazhan Zhang, Zhanchao Zhang, Dong Pu, Lulu Tang, Shikui Ma, Qiang Liu, Jing Dong, Kunlun He, Kunpeng Li, Da Teng, Tao Li

**Affiliations:** 1 Department of Medical Innovation Research Chinese PLA General Hospital Beijing China; 2 Dataa Robotics Co, Ltd Beijing China; 3 Department of Rheumatology and Immunology First Medical Center Chinese PLA General Hospital Beijing China; 4 Beijing Institute of Petrochemical Technology Beijing China

**Keywords:** spondyloarthritis, benchmark, large language model, artificial intelligence, AI, AI chatbot, AI-assistant diagnosis

## Abstract

**Background:**

Spondyloarthritis (SpA), a chronic inflammatory disorder, predominantly impacts the sacroiliac joints and spine, significantly escalating the risk of disability. SpA’s complexity, as evidenced by its diverse clinical presentations and symptoms that often mimic other diseases, presents substantial challenges in its accurate diagnosis and differentiation. This complexity becomes even more pronounced in nonspecialist health care environments due to limited resources, resulting in delayed referrals, increased misdiagnosis rates, and exacerbated disability outcomes for patients with SpA. The emergence of large language models (LLMs) in medical diagnostics introduces a revolutionary potential to overcome these diagnostic hurdles. Despite recent advancements in artificial intelligence and LLMs demonstrating effectiveness in diagnosing and treating various diseases, their application in SpA remains underdeveloped. Currently, there is a notable absence of SpA-specific LLMs and an established benchmark for assessing the performance of such models in this particular field.

**Objective:**

Our objective is to develop a foundational medical model, creating a comprehensive evaluation benchmark tailored to the essential medical knowledge of SpA and its unique diagnostic and treatment protocols. The model, post-pretraining, will be subject to further enhancement through supervised fine-tuning. It is projected to significantly aid physicians in SpA diagnosis and treatment, especially in settings with limited access to specialized care. Furthermore, this initiative is poised to promote early and accurate SpA detection at the primary care level, thereby diminishing the risks associated with delayed or incorrect diagnoses.

**Methods:**

A rigorous benchmark, comprising 222 meticulously formulated multiple-choice questions on SpA, will be established and developed. These questions will be extensively revised to ensure their suitability for accurately evaluating LLMs’ performance in real-world diagnostic and therapeutic scenarios. Our methodology involves selecting and refining top foundational models using public data sets. The best-performing model in our benchmark will undergo further training. Subsequently, more than 80,000 real-world inpatient and outpatient cases from hospitals will enhance LLM training, incorporating techniques such as supervised fine-tuning and low-rank adaptation. We will rigorously assess the models’ generated responses for accuracy and evaluate their reasoning processes using the metrics of fluency, relevance, completeness, and medical proficiency.

**Results:**

Development of the model is progressing, with significant enhancements anticipated by early 2024. The benchmark, along with the results of evaluations, is expected to be released in the second quarter of 2024.

**Conclusions:**

Our trained model aims to capitalize on the capabilities of LLMs in analyzing complex clinical data, thereby enabling precise detection, diagnosis, and treatment of SpA. This innovation is anticipated to play a vital role in diminishing the disabilities arising from delayed or incorrect SpA diagnoses. By promoting this model across diverse health care settings, we anticipate a significant improvement in SpA management, culminating in enhanced patient outcomes and a reduced overall burden of the disease.

**International Registered Report Identifier (IRRID):**

DERR1-10.2196/57001

## Introduction

Spondyloarthritis (SpA) is a chronic inflammatory disease that comprises a collection of diseases rather than a singular specific disease. It is commonly classified into several subtypes, including ankylosing spondylitis, psoriatic arthritis, reactive arthritis, enteropathic arthritis, juvenile chronic arthritis, and undifferentiated SpA. Among these subtypes, ankylosing spondylitis is the most prevalent, characterized by radiologically positive axial SpA [1].

These subtypes of SpA often have many similar clinical, radiological, and serologic features, and primarily affect the axial skeleton and sacroiliac joints, resulting in a decline in spinal function and a significant impairment of daily life due to persistent back pain and arthritic complications. Early and comprehensive management of the disease is crucial to alleviate the symptoms, improve the quality of life, and reduce the risk of severe complications [2]. As of 2019, there are more than 200 million individuals afflicted with rheumatic diseases in China. Yet, there is a pronounced disparity between the number of these patients and the rheumatology and immunology specialists available to treat them. The shortage of dedicated professionals is compounded by the absence of specialized departments in many hospitals; fewer than one-third of tertiary hospitals nationwide have independent rheumatology and immunology departments. This disparity is even more acute in remote clinics with limited resources, where the deployment of trained, validated diagnostic assistive models could be instrumental in facilitating early diagnosis and appropriate treatment recommendations, thus diminishing the broader impact of the disease. Currently, the integration of large language models (LLMs) within rheumatology, specifically tailored to SpA, remains unexplored.

Foundation models [3], once refined with domain-specific data sets, exhibit remarkable capabilities in specialized fields. These models adeptly execute diverse tasks, including sentiment analysis, image captioning, and command execution. The health care sector has seen a notable increase in chatbot usage, where they provide advanced, personalized treatment guidance. Enhanced with comprehensive medical data sets, foundation models proficiently interpret and articulate intricate medical terminologies while offering reliable question-answer services. LLMs, such as generative pretrained transformers, are recognized as foundational models and are increasingly being used in medical settings, owing to their exceptional ability to process and generate natural language.

In the realm of medicine, LLMs exhibit significant potential across clinical, educational, and research domains. They play a crucial role in enhancing clinical diagnostics and treatments, advancing physician training, and fostering robust clinical research within health care organizations [4]. Purpose-built LLMs such as foresight, effectively predict outcomes using electronic health records [5]. Novel architectures, such as the hybrid value-aware transformer, enhance the performance of LLMs by integrating longitudinal and multimodal clinical data [6]. Additionally, LLMs excel in tasks that extend beyond expert domains or are guided by user prompts [7-9], thereby, significantly reducing physicians’ administrative load, as seen in the preparation of discharge summaries. However, challenges including accuracy, outdated training data, the potential for producing plausible but incorrect information, lack of transparency, and ethical issues persist. Despite these challenges, the development of LLMs continues, focusing on enhancing their efficacy and safety in health care applications.

In the health care sector, particularly within rheumatology, there exists a potential for advanced models, such as ChatGPT (OpenAI), to produce incorrect outputs or disseminate medically unsound information, masquerading as authoritative advice [10]. This risk is exacerbated in conditions like SpA—a disease prone to diagnostic challenges and frequent misidentification—where a lack of comprehensive evaluation by LLMs prevails. Evaluation methodologies often resort to subjective measures. Kung et al [11] introduced the accuracy, concordance, and insight metrics, while Uz and Umay [10] developed the reliability and usefulness scores for appraising ChatGPT’s responses. Gilson et al [12] assessed ChatGPT’s response quality via logical reasoning and the relevance of internal and external information.

The following benchmarks collectively underscore the diverse methodologies and applications within medical artificial intelligence (AI), ranging from language understanding to clinical decision-making. The Comprehensive Medical Benchmark in Chinese (CMB) [13] and the Chinese Biomedical Language Understanding Evaluation Benchmark (CBLUE) [14] are notable for their focus on linguistic and cultural nuances in the medical context. CMB evaluates LLMs like ChatGPT and GPT-4 (OpenAI) within the framework of traditional Chinese medicine, reflecting the importance of cultural context in medical AI. Similarly, CBLUE assesses pretrained Chinese models for biomedical language understanding, emphasizing the need for language-specific AI tools in health care. Chen et al’s [15] work on a benchmark for automatic medical consultation systems represents a leap toward practical AI applications in health care. By proposing frameworks for doctor-patient dialog understanding and task-oriented interaction, this research paves the way for AI to enhance patient engagement and clinical workflows. MedEval [16] stands out for its comprehensive approach, encompassing a multilevel, multitask, and multidomain medical benchmark. This benchmark integrates data from various health care systems, covering an extensive range of medical tasks and scenarios, thereby demonstrating the versatility required in medical AI applications. Recent additions to this array of benchmarks include an explainable and reliable medical report generation benchmark based on fundus fluorescein angiography images and reports [17], which focuses on reliability and explains the ability of medical report generation, a critical aspect in clinical documentation and patient care.

In response to the absence of a standardized benchmark within rheumatology, Madrid-García et al [18] have made a significant contribution by creating a data set comprising 145 rheumatology-related questions, meticulously extracted from the Spanish Médico Interno Residente exams conducted between the academic years 2009-2010 and 2022-2023. This data set is thoughtfully categorized into “Case” and “Factual” types, including 14 questions that are specifically related to SpA. In these benchmarks, there are few questions related to SpA, and the variety is also limited, which hinders the accurate and objective assessment of LLMs’ accuracy in addressing the queries related to SpA. In collaboration with clinical experts, we are in the process of developing a benchmark data set consisting of 222 multiple-choice questions on SpA. This initiative aims to challenge models to demonstrate a profound understanding of medical knowledge and advanced reasoning skills. The questions span various degrees of difficulty and formats, encompassing both medical concepts and case-based scenarios. Assessments bifurcate into answer-only and chain-of-thought (CoT) approaches. We prioritize model accuracy while also using fluency, relevance, completeness, and proficiency in medicine from the CMB benchmark’s metric to gauge the quality of the model’s response.

We aim to rigorously assess the foundation models by refining them with publicly accessible medical databases and a bespoke data set comprising authentic medical cases, culminating in the development of our proprietary model, RobotGPT (Dataa Robotics Inc). Our goal is to augment their adeptness in fielding SpA-centric inquiries. Forthcoming experiments, as outlined in the Methods section, will benchmark the efficacy of various LLMs against our novel spondyloarthritis multi-choice question answering (SpAMCQA) data set. Furthermore, we aspire to juxtapose our model’s explanatory capabilities with its peers by using fluency, relevance, completeness, and proficiency in medicine as our evaluative metric.

## Methods

### Benchmark

#### Overview

The development of a robust benchmark is crucial for the advancement of LLMs in the medical domain, particularly in specialized domains not adequately addressed in the existing literature. The section “Setting” of our protocol unveils a thoroughly developed benchmark dedicated to SpA, addressing a significant gap in available data sets and enabling a comprehensive evaluation of LLMs in this specialized field. The section “Evaluation Framework” outlines the evaluation framework for these questions, highlighting the data set’s reliability and value in precisely evaluating the diagnostic and therapeutic reasoning abilities of LLMs.

#### Setting

Current medical benchmark data sets encompass an extensive array of medical subjects, but there is a notable deficiency regarding SpA. To address this shortfall, our team has developed an innovative bilingual (English and Chinese) benchmark data set comprising multiple-choice questions specifically about SpA, designed to objectively measure the precision of LLMs.

Knowledgeable clinicians from the Department of Rheumatology and Immunology at Chinese People’s Liberation Army (PLA) General Hospital gathered insights on SpA from essential medical literature [19-21]. Capitalizing on their profound clinical experience and informed by established question banks, they created 222 multiple-choice questions. These questions were meticulously refined through multiple rounds of expert review, guaranteeing their lucidity and precision. Spanning various levels of complexity, they simulate genuine diagnostic and therapeutic situations, thus offering a comprehensive measure to appraise the capability of LLMs.

The benchmark data set consists of 194 single-answer and 28 multiple-answer questions, categorized into 106 questions focused on medical knowledge and 116 questions on case analysis. The experts categorized them by types (concept definition, logical inference, and case analysis), dimensions (etiology, clinical manifestations, diagnosis, treatment, and multidimensional), themes (exclusively SpA and SpA alongside other diseases), and complexities (elementary, intermediate, and advanced), according to their professional judgment. These classifications are thoroughly itemized in Table 1.

**Table 1:** Comprehensive overview of the benchmark data set tailored for SpA^a^ in the evaluation of LLMs^b^.

Question categories		Single-choice questions (n=194), n	Multiple-choice questions (n=28), n	Total (N=222), n
**Type**				
	Concept definition	49	13	62
	Logical inference	33	11	44
	Case analysis	112	4	116
**Dimensions**				
	Etiology	16	0	16
	Clinical manifestations	26	7	33
	Investigation	14	7	21
	Diagnosis and differential diagnosis	114	7	121
	Treatment	12	4	16
	Multidimensional	12	3	15
**Theme**				
	Exclusively SpA	75	21	96
	SpA alongside other diseases	119	7	126
**Complexities**				
	Elementary	93	5	98
	Intermediate	70	13	83
	Advanced	31	10	41

^a^SPA: spondyloarthritis.

^b^LLM: large language model.

#### Evaluation Framework

##### Overview

**Figure 1:**
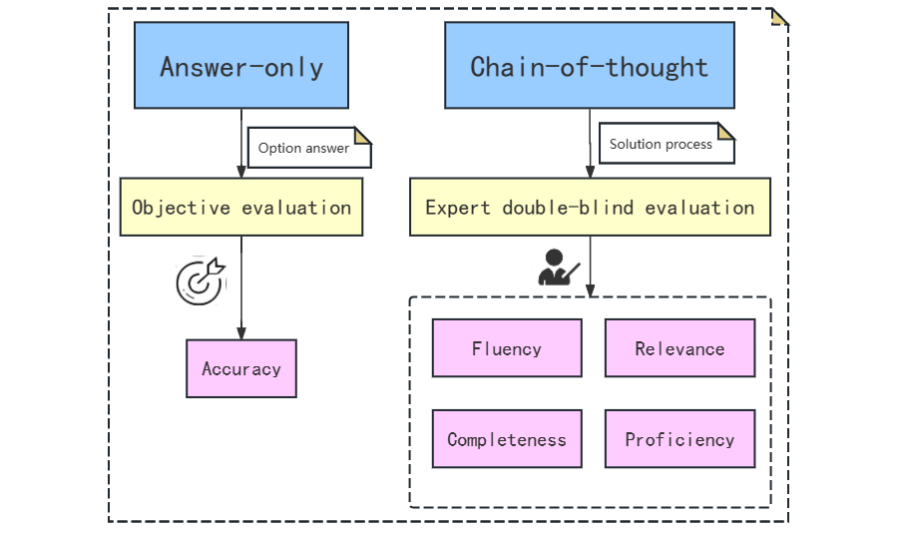
Evaluation framework for assessing LLMs in SpA diagnosis and treatment. LLM: large language model; SpA: spondyloarthritis.

Figure 1 presents the SpAMCQA experimental setup. Our evaluation outlines 2 strategies—answer-only and CoT. CoT prompting and guiding LLMs through sequential reasoning, has improved problem-solving across tasks. By using CoT, we investigated LLMs’ performance in both answer-only and CoT contexts [22]. Responses to multiple-choice questions were quantitatively evaluated using objective criteria while rating each response across 4 aspects—fluency, relevance, completeness, and medical proficiency—using a grading scale from 1 to 5. This 2-fold methodology ensures an exhaustive evaluation of LLMs, gauging both accuracy and analytical depth.

##### Objective Evaluation

**Figure 2:**
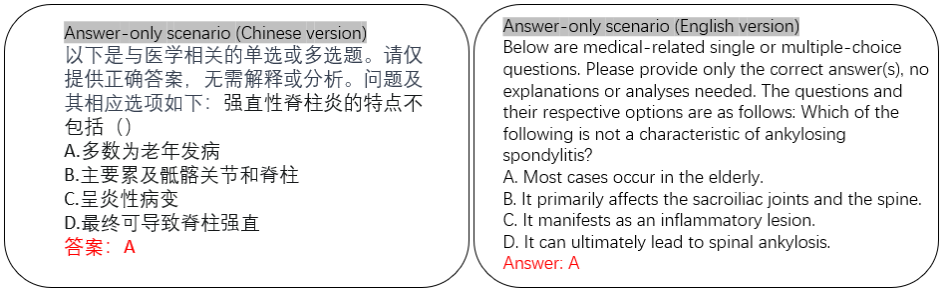
Answer-only evaluation setting for LLM assessment in SpA diagnosis (Chinese and English versions). It showcases the format and structure of multiple-choice questions designed specifically for this study, highlighting how models are required to select the correct answer without additional contextual cues or reasoning paths. LLM: large language model; SpA: spondyloarthritis.

Figure 2 illustrates the input prompts tailored for the answer-only evaluation. Examples illustrating comprehension tasks in answer-only scenarios are presented in both English and Chinese versions. The completed responses from LLMs are highlighted in red, whereas the inputted prompts are shown in black text. In this setup, the models must discern the correct answer without auxiliary cues. For single-choice queries, scoring depends solely on the correct selection. Multiple-choice question scoring adopts principles from the Chinese Medical Examinations (CMExam), where each question is worth 1 point, divided equally among the correct answers. Correct selections earn their share of points, while incorrect ones deduct an equivalent amount, down to 0. For example, in a question with 5 answer choices, 2 of which are correct, selecting 2 correct and 1 incorrect option yields a 0.5 score; omitting 1 correct option also results in a score of 0.5.

##### Expert Evaluation on CoT

**Figure 3:**
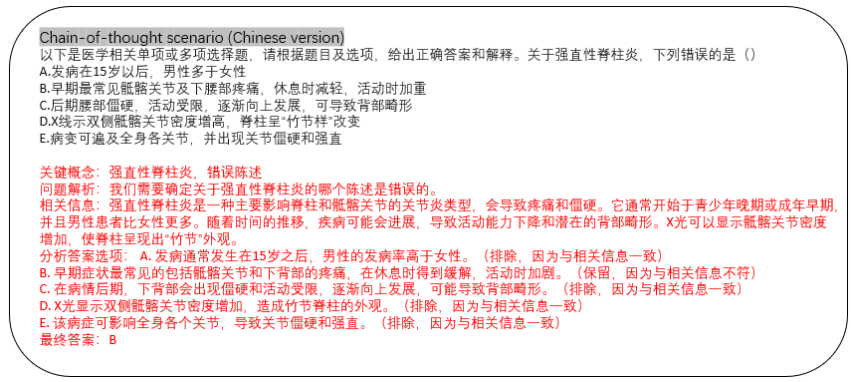
CoT evaluation setting for LLM assessment in SpA diagnosis (Chinese). Unlike the “Answer-Only” setting, the CoT approach requires the models not only to select the correct answer but also to provide a reasoned explanation for their choice, mimicking a clinician’s thought process in diagnosing SpA. CoT: chain-of-thought; LLM: large language model; SpA: spondyloarthritis.

**Figure 4:**
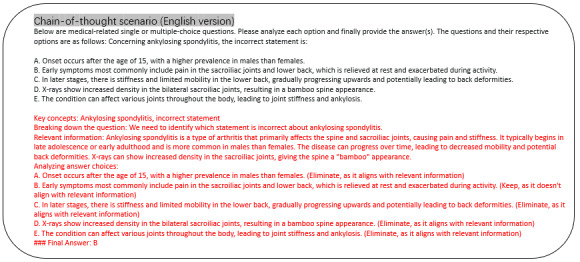
CoT evaluation setting for LLM assessment in SpA diagnosis (English). Unlike the “Answer-Only” setting, the CoT approach requires the models not only to select the correct answer but also to provide a reasoned explanation for their choice, mimicking a clinician’s thought process in diagnosing SpA. CoT: chain-of-thought; LLM: large language model; SpA: spondyloarthritis.

Merely providing an answer does not fully assess a model’s competence in processing medical queries, as it might rely on guesswork. In clinical practice, an expert critically evaluates the question and each answer choice. Accordingly, we require the model to present its selection with a corresponding CoT rationale, as depicted in Figures 3 and 4. Examples illustrating comprehension tasks in CoT scenarios are presented in both Chinese and English versions. The completed responses from LLMs are highlighted in red, while the inputted prompts are shown in black text. To evaluate the quality of the rationale, we conducted a double-blind study with 2 medical experts rating each response across 4 aspects.

### Modeling

#### Trial Design

The “Modeling” section delineates our approach to constructing and enhancing a specialized LLM for the medical field. It involves curating a comprehensive medical data set, selecting a cutting-edge foundation model, and applying rigorous fine-tuning methods to elevate performance. Initially, the data sets, both public and proprietary, which inform the model’s training will be described. Subsequently, a leading foundation model will be identified through empirical testing. Finally, our fine-tuning strategy, tailored to our medical benchmark, aims to improve the model’s precision and use in medical diagnostics and therapy.

#### Ethical Considerations

All experimental protocols were approved by the Ethics Review Committee of Chinese PLA General Hospital (S2022-255-01). This study adhered strictly to the hospital’s data management and usage protocols, applying deidentification procedures to patient-specific information such as names, national identification numbers, and medical record IDs within the extracted medical records to protect patient privacy. Additionally, researchers involved in this study signed confidentiality agreements, and the development of the model training and application systems was conducted entirely within the hospital’s internal network, effectively eliminating the risk of information breaches. However, the initial training of generic data was performed by technicians on external networks, using data sets that are publicly available. Throughout the research process, we diligently followed applicable laws, regulations, and standards to ensure that our research activities and data usage conformed to ethical principles and legal requirements.

#### Data Sets

The Huatuo-26M [23] data set, comprising 26 million medical question and answer (Q&A) pairs, is categorized into 4 distinct segments—medical descriptions generated by automated tools, as well as both simulated and authentic single-turn and multi-turn medical dialogs. It is versatile, aiding in training Q&A data sets, serving as a base for retrieval-augmented generation, and enhancing pretrained language models.

In the future, a collection of 100,000 multiple-choice questions will be compiled from diverse sources such as CMB, CMExam [24], MedQA [25], and MLEC-QA [26], with each source contributing 25,000 questions. Among these, only the CMExam data set includes explanations. For SpA and related rheumatic diseases, we generated explanations via ChatGPT, which were then validated by medical professionals. Additionally, SpA-focused questions from professional medical examinations will be incorporated into the training corpus, carefully avoiding duplication with the benchmark data set.

To refine the LLMs’ proficiency in multiple-choice question responses, we extracted Q&A pairs from academic publications and medical texts, thus expanding the model’s feature repertoire and enhancing its domain-specific knowledge. These Q&As, infused with rich contextual and semantic detail, are instrumental in advancing the model’s predictive precision.

Drawing on a data set encompassing more than 80,000 medical cases from the Chinese PLA General Hospital, we assembled a substantial corpus of more than 390,000 consultations. Table 2 presents the initial demographic distribution of patients by age and gender. We adhered to stringent privacy compliance protocols in refining the data into categories of symptoms, diagnoses, and treatment plans. The hospital’s medical records were meticulously prepared for model training by deduplicating data, correcting formatting inconsistencies, and anonymizing personal information to uphold patient confidentiality and meet privacy standards. To enhance the data set’s diversity, it was imperative to incorporate cases diagnosed with ankylosing spondylitis and those presenting similar symptoms, such as SpA, psoriatic arthritis, reactive arthritis, sacroiliac joint infection, hypophosphatemic rickets, lumbar disk herniation, and rheumatoid arthritis. These cases were stringently validated by medical experts to ensure their relevance to the diseases of interest. When the data set for a specific disease type exceeds 200 instances, we cap it at 200; if it contains fewer than 200, all instances are included in the training set. The training set was carefully curated to include approximately 1400 verified medical records, covering 8 distinct disease categories. The internal hospital record–based training corpus was formatted as single-choice diagnostic queries. Each query comprised patient demographics, clinical history, physical examination outcomes, and auxiliary test results, followed by a set of 4 options—1 correct answer and 3 distractors.

**Table 2:** Demographic distribution of patients with SpA^a^ in the study population^b^.

Age range at first diagnosis (years)	Males, n	Females, n
0-10	140	59
10-20	7263	1631
20-30	21,303	7140
30-40	16,389	7602
40-50	7837	4887
50-60	3301	2918
60-70	1129	995
70-80	283	240
80-90	84	66
90-100	10	2

^a^SpA: spondyloarthritis.

^b^The table details the demographic characteristics of patients at their initial consultation for SpA treatment, categorized by age and gender, within the study cohort.

#### Selection of the Foundation Model

From the C-Eval leaderboard [27], we selected a series of the latest open-source models—Baichuan-13B (Baichuan Inc), ChatGLM2-6B (Beijing Zhipu Huazhang Technology Co, Ltd), Llama2-7B (GenAI, Meta), Qwen-7B (Aliyun). Concurrently, we use a pretrained model RobotGPT, an LLM that integrates ideas from influential papers such as InstructGPT, LLaMA, and Bloom. This model was built on approximately 200 TB of raw, unfiltered data and underwent fine-tuning with about 3 TB of high-quality, meticulously curated data. The data set supporting RobotGPT is diverse and comprehensive, including data from various sources—25% from Common Crawl, 16% from the multilingual Open Super-large Crawled Aggregated Corpus (OSCAR) database, and 14% from books. Additionally, 8% of the data set comprises Chinese novels, enriching its linguistic diversity. The model also integrates a substantial amount of programming-related data, with 12% from GitHub Code and 13% from CodeContests Code. The remaining 12% of the data set consists of various multilingual sources, further enhancing the model’s robustness and versatility in processing different languages and contexts. RobotGPT is available in 13B and 34B versions.

We fine-tuned 6 state-of-the-art open-source models with different parameter versions on the public data set (in the section “Data Sets”)—ChatGLM2-6B-32K, Baichuan-13B-Chat, Llama2-7B-Chat, Qwen-7B-Chat, Qwen-14B-Chat, and Openbuddy-70B. After evaluating these fine-tuned models with 122 test questions sourced from public data, we found that Qwen-7B outperformed Qwen-14B, while the largest model, Openbuddy-70B, did not achieve significant results compared with the others. Therefore, only ChatGLM2-6B-32K, Baichuan-13B-Chat, Llama2-7B-Chat, and Qwen-7B-Chat were selected for further fine-tuning with EMR data.

#### Fine-Tuning

Supervised fine-tuning facilitates the enhancement of model performance on specific data sets by leveraging pretrained knowledge combined with minor task-specific adjustments. This is particularly critical in the medical field, where the acquisition and review of specialized textual data often incur high costs and are challenging to amass in large quantities. Supervised fine-tuning allows for superior model performance with fewer specialized data sets. Although this method can improve performance for specific tasks, it poses a risk of overfitting, especially when the training data are exceedingly limited. To address this, we constructed Q&A pairs and extracted examination questions and answers from publicly available data sets, and balanced data samples across various diseases within a private medical record data set to supervise the model fine-tuning with multiple-choice questions. We then applied low-rank adaptation (LoRA) [28] fine-tuning technique, freezing the pretrained model’s weight parameters while incorporating additional network layers without altering the original model parameters, training only the parameters of these new layers. Given the relatively small number of added parameters, this approach significantly reduces the cost of fine-tuning while achieving results comparable to the comprehensive model fine-tuning. This technique enables the model to use the general knowledge acquired during the pretraining phase and learn features useful for addressing the questions related to the diagnosis and treatment of SpA.

**Figure 5:**
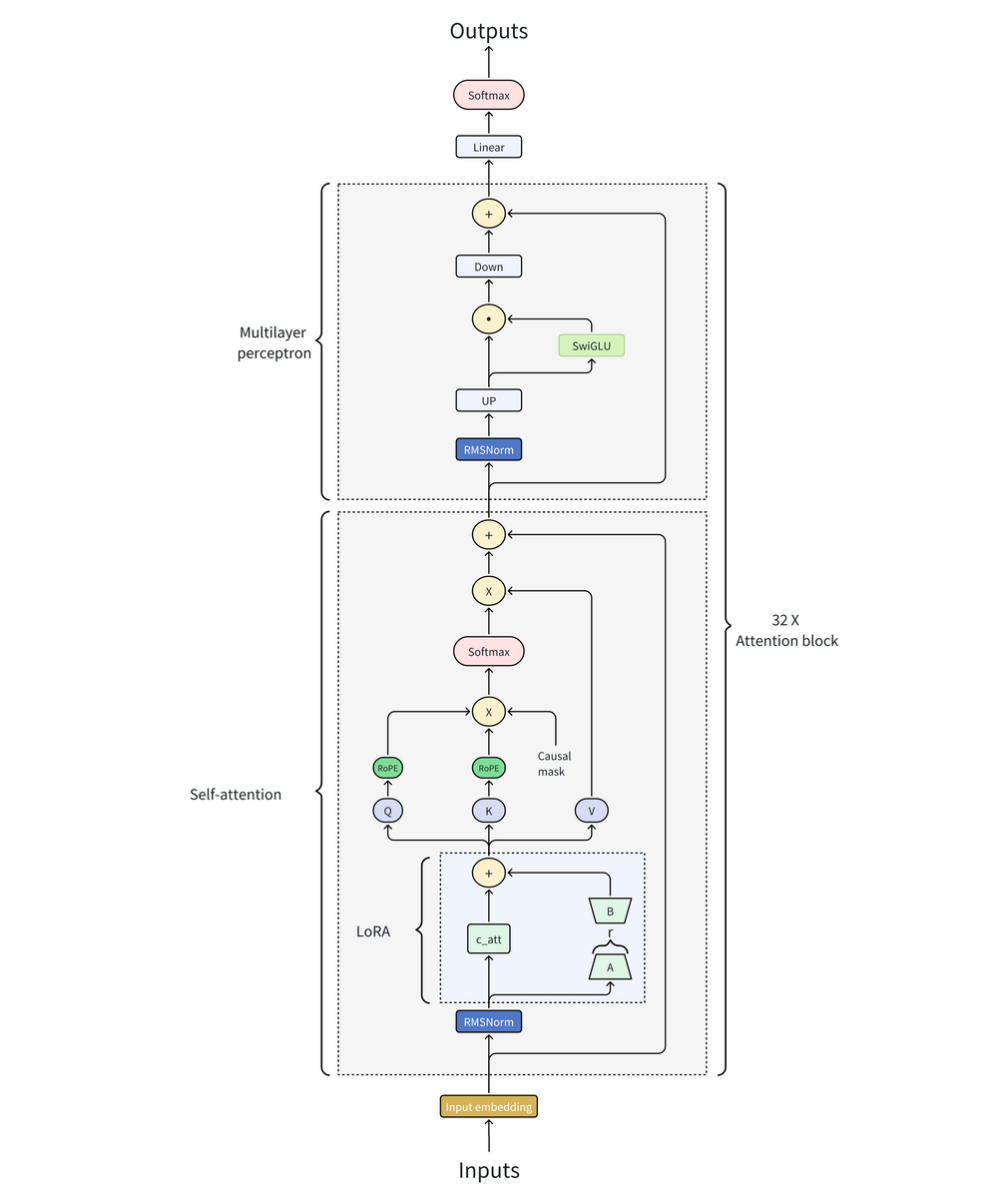
Neural network architecture with LoRA for LLMs in SpA diagnostics. LLM: large language models; LoRA: low-rank adaptation; SpA, spondyloarthritis.

LoRA involves integrating trainable rank decomposition matrices into transformer layers while preserving the pretrained weights. A significant advantage of LoRA is its ability to drastically reduce the number of trainable parameters by a factor of 10,000, and to decrease graphic processing unit memory usage by 3-fold. Figure 5 shows the neural network model implementing LoRA for advanced fine-tuning. The model’s architecture features an input embedding module, a self-attention mechanism with query (Q), key (K), value (V) elements, a Softmax layer, and a causal mask. Additionally, it includes a multilayer perceptron, specialized components like Swish gated linear unit and root mean square layer normalization, and concludes with a linear and Softmax layer for generating predictions. This strategic enhancement will be applied to a Q&A data set, sourced from the extensive medical consultation records at Chinese PLA General Hospital.

## Results

At the time of paper submission, our advanced medical model, RobotGPT-30B, ranks first on the CMB leaderboard. The extraction of internal medical record data from the hospital has been completed, and the selection of the base model has been finalized. Model development is underway, with further development planned for early 2024. The benchmark will be released in May 2024. The outcomes of both objective and subjective evaluations are scheduled to be published in June 2024. This project was funded in December 2021 and concluded in November 2024.

## Discussion

### Limitations

In the domain of health care, LLMs may encounter inherent limitations when addressing the nuances of specific medical fields. This is particularly evident in scenarios involving rare diseases or unique treatment protocols, where these models may fail to deliver accurate diagnostics. A notable challenge for these models, especially when parsing complex medical literature and data, is the propensity for “hallucinations”—the generation of seemingly plausible but unfounded or erroneous assertions in the absence of sufficient evidence. This issue is of paramount importance in clinical settings, where it could significantly impact the quality of diagnoses and treatments.

### Continuous Enhancement and Future Work

As the corpus of medical knowledge continues to expand and clinical practices advance, the diagnostic and treatment guidelines for SpA are subject to frequent updates. In response, we have established a systematic framework for the periodic review and enhancement of our LLMs to maintain their efficacy and accuracy within clinical applications. We envisage conducting a comprehensive evaluation of the model biannually to ascertain the necessity for updates, although this interval may be adjusted in the light of significant breakthroughs in the medical field or the release of new guidelines. Upon identifying the need for an update, our team will initiate the collection of pertinent new data, including recent research publications, clinical trial outcomes, and updated treatment guidelines. These data will be scrutinized by clinical experts before technical personnel proceed with the fine-tuning of the existing model using the validated data set, making requisite adjustments and optimizations to ensure our model accurately reflects the latest knowledge and guidelines. We believe that through continuous updates and enhancements, our language model will become an indispensable tool for clinicians in the diagnosis and treatment of SpA.

To ensure a thorough assessment, we plan to broaden the benchmark data set to include a wide array of questions. Our long-term objective is to expand the benchmark data set to cover a comprehensive range of major disease categories. The preliminary results of our model are promising, and we foresee further enhancements through improvements in data quality, volume, and model architecture. For optimal performance, we advise future studies to include a broader range of real clinical data and to incorporate demographic factors such as gender and age for more nuanced model insights. Although our current focus is on SpA, our methodology is designed to be adaptable to other diseases. We are also exploring the potential of expanding our research into the multimodal domain, including medical image analyses such as x-ray and magnetic resonance imaging. This integrated approach, combining textual and visual data, aims to transform patient diagnosis and treatment, with a view toward potential application in medical robotics. These robots, equipped with computer vision, will be able to interpret medical images and, complemented by insights from the language model, offer preliminary diagnoses. With the ongoing enhancement of capabilities in LLM technologies, medical models specialized for vertical domains are anticipated to soon integrate with the health care information systems such as hospital information systems and laboratory information systems. This synergy is expected to significantly improve the diagnostic and therapeutic efficiency of health care facilities, heralding a paradigmatic shift in medical diagnostics. For instance, by leveraging LLMs to directly access patients’ laboratory tests, imaging data, and historical medical records, it is possible to recommend treatment plans to the attending physicians. This approach has the potential to significantly enhance diagnostic and therapeutic efficiency while conserving health care resources.

### Conclusions

Our main findings are expected to reveal that the RobotGPT model, after fine-tuning, will exhibit promising capabilities in navigating the intricate landscape of SpA diagnosis and treatment recommendations, particularly in resource-limited settings where specialized care is scarce. Simultaneously, our benchmark and evaluation methods will be well-equipped to measure the capabilities of LLMs in diagnosing and treating SpA issues.

Our approach builds upon the foundational work in LLMs, extending their application to a niche yet critically important area of medicine. Upon fine-tuning with domain-specific public and proprietary data sets, the capacity of LLMs to address SpA inquiries is anticipated to enhance significantly. Concurrently, our model will be evaluated against state-of-the-art counterparts through both objective accuracy and subjective assessments, with the expectation that our model will achieve commendable performance. While benchmarks like CMB [13], CBLUE [14], and MedEval [16] have been instrumental in advancing LLMs in general medical contexts, the novelty of our approach lies in the development of a tailored benchmark for SpA, addressing the gap in existing LLM applications within rheumatology. This benchmark not only facilitates the accurate assessment of the model’s performance but also ensures its relevance to real-world clinical scenarios.

Through continuous updates and enhancements, our language model will become an indispensable tool for clinicians in the diagnosis and treatment of SpA. Simultaneously, the development of this benchmark for LLMs in the context of SpA establishes a new precedent in rheumatology research. It serves not only to evaluate the accuracy of AI-driven diagnostics in this specific disease area but also acts as a prototype for comparable advancements in other rheumatological conditions.

## Abbreviations

AI: artificial intelligence
CBLUE: Chinese Biomedical Language Understanding Evaluation Benchmark
CMB: Comprehensive Medical Benchmark
CMExam: Chinese Medical Examinations
CoT: chain-of-thought
LLM: large language model
LoRA: low-rank adaptation
OSCAR: Open Super-large Crawled Aggregated Corpus
PLA: People’s Liberation Army
Q&A: question and answer
SpA: spondyloarthritis
SpAMCQA: spondyloarthritis multi-choice question answering

## Data Availability

The data sets from the public data sets analyzed in this study are available in the CMB [13], Huatuo-26M [23], CMExam [24], MedQA [25], and MLEC-QA [26]. Additionally, the data sets from the private data sets trained and generated during this study are not publicly available due to protect patient privacy but are available from the corresponding author on reasonable request.
